# Increased spatial coherence length from an asymmetric crystal reflection at grazing exit

**DOI:** 10.1107/S1600577524001425

**Published:** 2024-03-26

**Authors:** Albert Macrander, Xianbo Shi, Walan Grizzoli, Xianrong Huang, Nino Pereira, Michael Wojcik, Lahsen Assoufid

**Affiliations:** a Advanced Photon Source, Argonne National Laboratory, Argonne, IL 60439, USA; b Ecopulse, Inc., 7844 Vervain Ct, Springfield, VA 22152, USA; RIKEN SPring-8 Center, Japan

**Keywords:** X-ray crystal asymmetric diffraction, grazing exit, spatial coherence length

## Abstract

Coherent X-ray imaging is an active field at synchrotron sources. The images rely on the available coherent flux over a limited field of view. At many synchrotron beamlines a double-crystal monochromator (DCM) is employed in a standard nondispersive arrangement. For coherent diffraction imaging it is advantageous to increase the available field of view by increasing the spatial coherence length (SCL) of a beam exiting such a DCM. Here, Talbot interferometry data together with ray-tracing simulations for a (+ − − +) four-reflection experimental arrangement are presented, wherein the first two reflections are in the DCM and the final fourth reflection is asymmetric at grazing exit. Analyses of the interferometry data combined with the simulations show that compared with the beam exiting the DCM a gain of 76% in the SCL was achieved, albeit with a factor of 20 reduction in flux density, which may not be a severe penalty at a synchrotron beamline. Previous efforts reported in the literature to increase the SCL that employed asymmetric crystal diffraction at grazing incidence are also discussed. A much reduced SCL is found presently in simulations wherein the same asymmetric crystal is set for grazing incidence instead of grazing exit. In addition, the present study is compared and contrasted with two other means of increasing the SCL. These are (i) focusing the beam onto an aperture to act as a secondary source, and (ii) allowing the beam to propagate in vacuum an additional distance along the beamline.

## Abstract 2

A multiple crystal diffraction arrangement with a final asymmetric crystal diffracting at grazing exit was found to yield a 76% longer spatial coherence length compared with that for a beam directly exiting a double-crystal monochromator.

## 1. Introduction

Coherent X-ray imaging is an active field at synchrotron sources that employs the available coherent flux over a limited field of view (Nugent, 2010 ▸; Jacobsen, 2020 ▸). The field of view that can be imaged is limited by the spatial coherence length (SCL). For these studies it is often advantageous to have a field of view as large as possible so that larger samples can be imaged coherently. The present work details measurements by which the SCL was increased over that available directly from the beamline double-crystal monochromator (DCM) at beamline 1-BM, a bending magnet beamline at the Advanced Photon Source (APS), USA.

We report simulations that demonstrate that the spatial coherence length can be increased significantly for an asymmetric Si 111 Bragg reflection at grazing exit. A four-reflection (+ − − +) diffraction arrangement was employed for which the first two reflections were in the DCM. The beam from the DCM was first diffracted dispersively from a symmetric Si(111) crystal and then diffracted from an asymmetric Si 111 Bragg reflection at grazing exit. The asymmetry angle built into the crystal was 13.1°. Talbot measurements for the coherence length were obtained at APS beamline 1-BM (Marathe *et al.*, 2014 ▸; Macrander *et al.*, 2016 ▸) at 8.500 keV. A value of 39.8 µm was measured for the SCL. For the beam directly exiting the DCM a value of 22.6 µm was measured. In addition, extensive ray tracing (Rebuffi & Sanchez del Rio, 2016 ▸, 2017 ▸) simulations simulate the data well. At grazing exit the effective source is much reduced in size and was found to deviate from a Gaussian profile. The effective source was found to be only 3.2 µm FWHM, a quite small value. Ray-tracing simulations with the effective source are presented. The effects of wavelength dispersion were included automatically in the ray tracing. We report that the grazing exit geometry can indeed increase the SCL significantly over that for a beam exiting a DCM.

Asymmetric Bragg diffraction at grazing incidence instead of at grazing exit has previously been considered to improve the spatial coherence of synchrotron beams. Collimation is achieved in a grazing incidence geometry, and since the SCL is inversely proportional to a divergence, albeit at a specific point in the object field, a longer SCL might be achieved. In general, there is the complication of chromatic aberration, that is wavelength dispersion, arising from a finite bandwidth which increases the output divergence from an asymmetric crystal. Brauer *et al.* (1995*a* ▸) report that the grazing incidence geometry is severely plagued by such chromatic aberration. The work by Ishikawa (1988 ▸) reports an SCL of 220 µm, albeit for a final monochromatic beam with a divergence of only 0.04 µrad achieved after two sequential asymmetric reflections at grazing incidence. The sample in their case was a wedged crystal studied in a non-dispersive Laue geometry. This non-dispersive arrangement cleverly countered the effect of chromatic aberration. However, a required experimental arrangement that mitigates the effects of chromatic aberration complicates the general usefulness of the double grazing incidence geometry reported by Ishikawa. See Appendix *A* [App appa] for comments on a grazing incidence geometry for the fourth crystal in our experimental arrangement. See Appendix *B* [App appb] for a comparison of the present method with other methods to increase the SCL.

Somewhat counter-intuitively, a reverse or grazing exit geometry was found presently to yield a significantly longer SCL than was available directly from the DCM. We note that a grazing exit geometry has been studied previously with the intent to improve the coherent flux through a pinhole with a diameter of a few micrometres (Brauer *et al.*, 1995*b* ▸). The present study was instead focused on simply increasing the spatial coherence length for the purpose of coherent diffraction imaging studies. This was found to be possible, with a gain of 76% over the SCL of the beam directly exiting the DCM, albeit with a factor of 20 reduction in flux density.

## 2. Experimental layout

**Figure 1:**
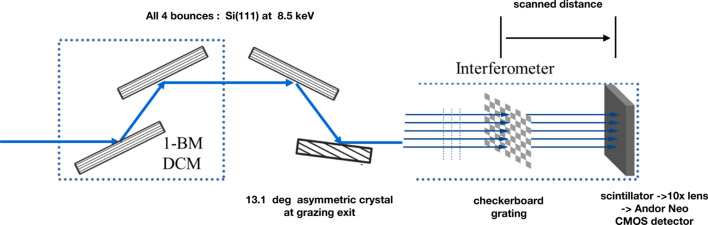
Experimental layout with the beam from the APS 1-BM bending magnet incident from the left. The first two crystal reflections were in the beamline monochromator. All four crystals were set to diffract in the vertical plane. For the final asymmetric crystal the grazing exit angle at 8.500 keV was 0.41°. The Talbot interferometer consisted of a checkerboard grating with period of 4.8 µm and located 35.5 m downstream of the bending magnet together with an Andor Neo CMOS detector having a pixel size of 6.5 µm. The detector recorded visible-light images from a LuAG:Ce scintillator magnified by a 10× lens to yield a 0.65 µm per pixel resolution. The asymmetric crystal was located 3.32 m upstream of the grating. Several hundred images were obtained at scanned distances between the grating and the scintillator out to 900 mm.

Fig. 1 ▸ shows a schematic layout in side view at beamline 1-BM of the APS that was used for the present measurements. The white-beam radiation from the bending magnet was apertured by slits to 3.0 mm vertically by 3.0 mm horizontally. The two silicon crystals in the DCM were set for the 111 reflection in a classic (+ −) geometry to produce a beam parallel to the main axis of the beamline. The first crystal was water cooled (Lang *et al.*, 1999 ▸). The physical distance between the bending magnet source and the grating was 35.5 m. However, for the beam directly from the DCM the source distance obtained from the Talbot interferometry in the vertical plane was 6 m shorter, and this could be simulated with a heat bump on the first crystal of the DCM (see below). The heat load of 1 W was such that a heat bump on the first crystal was expected. This heat bump was approximately 20 nm in height as estimated from wiggler results for a 1 W heat load at beamline ID-19 at the European Synchrotron Radiation Facility (Rutishauser *et al.*, 2013 ▸). Relevant changes made after the published description of the beamline (Macrander *et al.*, 2016 ▸) were: (i) removal of X-ray beryllium windows upstream of the DCM in conjunction with implementation of differential pumping such that the vacuum space in the DCM was contiguous with that in the storage ring, and (ii) installation of polished beryllium windows of 500 µm total thickness placed downstream of the DCM.

Downstream of these windows, two more silicon crystals were configured in a sequential arrangement (Stoupin *et al.*, 2016 ▸). The first of these was a 111 oriented silicon crystal set to diffract dispersively in a (+ − −) geometry as shown in Fig. 1 ▸. The second downstream crystal (the fourth crystal overall) was a silicon crystal with 111 planes oriented at 13.1° with respect to the surface. This final crystal was set to diffract at grazing exit. The exit angle at 8.5 keV was 0.41° and at 8.2 keV was 0.88°. The overall diffraction geometry for the four-crystal diffraction arrangement was (+ − − +).

A benefit of this four-crystal optical arrangement is a reduced bandpass. The bandpass after the third crystal was 0.8 eV FWHM and 0.2 eV after the fourth crystal. The beam incident on the fourth crystal corresponds to a longitudinal coherence length, λ^2^/(Δλ), equal to 2.8 µm. This value exceeds the extinction length of the asymmetric reflection which is 0.7 µm. We conclude that the longitudinal coherence length is not limiting presently, a conclusion further supported by the agreement we achieved between our *SHADOW* simulations and our Talbot interferometry measurements.

A Talbot interferometer based on a checkerboard pure phase grating was located downstream at a distance of 3.32 m from the asymmetrically diffracting crystal. The period of the checkerboard grating was 4.8 µm (Shi *et al.*, 2022 ▸). Alternating square areas of the grating were gold plated to a thickness of 1.62 µm. This provided a phase shift of π at the design energy for the grating of 8.0 keV. Very small phase shift differences at 8.2 keV and 8.5 keV did not affect the present analyses. Similarly, transmission factors differing from the design value of 0.52 at 8.0 keV up to 0.57 at 8.5 keV did not affect the present results, a finding attributable to the procedure of applying the second-order Fourier transform factor normalized to the zeroth order (Shi *et al.*, 2022 ▸). A LuAG:Ce X-ray scintillator which was imaged with 10× lens onto an Andor Neo CMOS detector was situated on a linear slide. The pixel size of the detector was 6.5 µm, which when combined with the 10× lens yielded a resolution of 0.65 µm for the X-ray patterns incident on the scintillator.

For image data the linear stage was scanned downstream along the beamline axis, labeled the *z*-axis, out to 900 mm in either 2 or 4 mm steps. Data were obtained not only for the four-reflection geometry but also for a beam exiting directly from the DCM, that is, for a two-reflection geometry. This served both to demonstrate the increased SCL attainable compared with the two-reflection geometry and to compare flux densities, since the four-reflection geometry comes at the cost of a reduced flux density.

## 3. Data processing

The image data were processed by *wavepy2* (*WavePy2.0*, https://github.com/APS-XSD-OPT-Group/wavepy2). The processing yielded points as a function of *z* for fringe visibilities along both vertical and horizontal directions. The visibility oscillates with a decreasing maximum on a length scale equal to one-eighth of the Talbot distance. The Talbot distance is given by 2*p* ^2^/λ, where *p* is the period (= 4.8 µm). The Talbot distance at 8.500 keV was 316 mm. The function by which the maxima decrease is known in the literature as the complex coherence factor (CCF) (Pfeiffer *et al.*, 2005 ▸), where the actual applied factor is the absolute value of a complex quantity. For an incoherent source the CCF can be calculated by means of the van Cittert–Zernike theorem (Goodman, 1985 ▸). The processing of the measured data proceeded by taking the Fourier transform and equating the amplitude of the second order, normalized by the zeroth order, to a visibility. As well, *wavepy2* yields a fit to the visibility with a Gaussian function. However, a Gaussian function was not assumed presently. The CCF was instead calculated from an effective source obtained by ray tracing to which the van Cittert–Zernike theorem was then applied to yield a simulated CCF.

## 4. Flux density comparison

**Figure 2:**
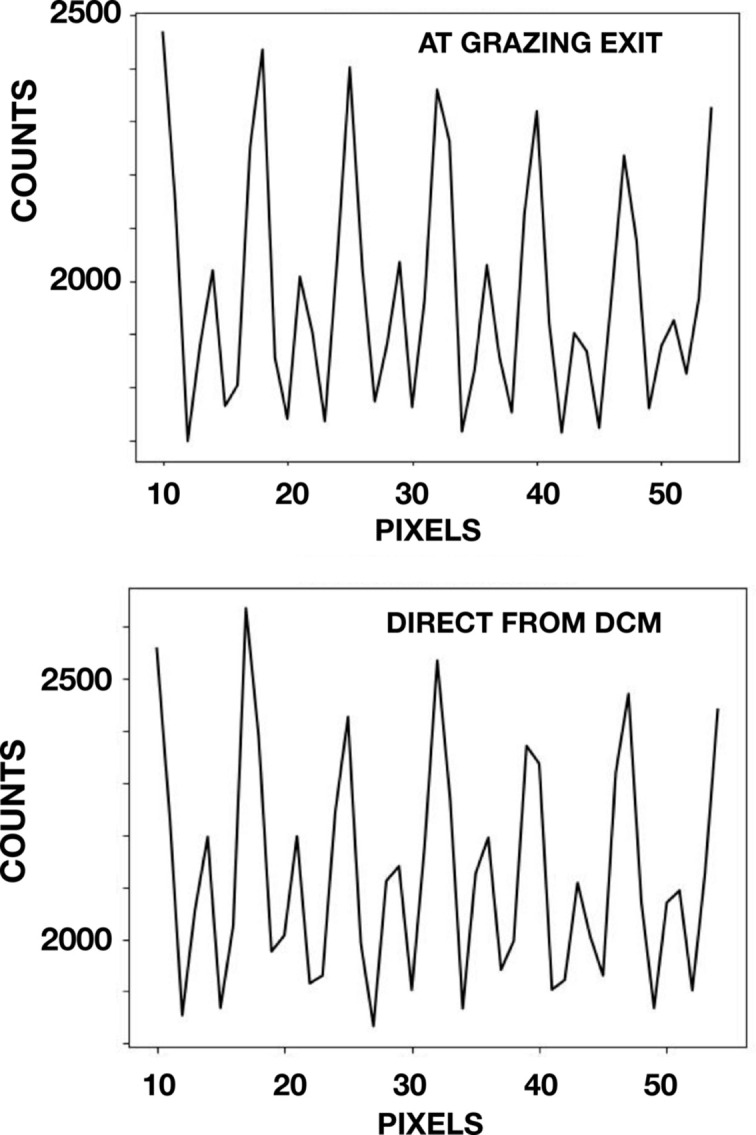
Flux density comparison at 8.500 keV and *z* = 20 mm downstream of the grating. In the upper panel is shown a crosscut profile of the counts per pixel recorded for the four-reflection geometry for a counting time of 120 s. In the lower panel a crosscut profile is shown for the two-reflection geometry for a counting time of 6 s. These data show that the flux density in the four-reflection geometry is 5% of that for the two-reflection geometry.

Fig. 2 ▸ shows crosscuts of detector images for both the four-reflection and the two-reflection geometries. For the four-reflection case the counting time was 120 s per image, whereas the counting time for the two-reflection geometry was 6 s. The images were obtained at *z* values of 1/16 of the Talbot distance. At 1/16 of the Talbot distance, and odd multiples thereof, the second-order Fourier coefficient of a crosscut profile is at a maximum. This is conveniently seen by examining the so-called ‘Talbot carpet’ of a π phase grating (Weitkamp *et al.*, 2006 ▸). The profiles in Fig. 2 ▸ are for six periods of the grating. For each period there are two peaks. One peak is weaker owing to absorption in the gold squares.

For the four-reflection geometry, the wavelength–angle phase space region on a Dumond diagram is reduced in comparison with that of two-reflection geometry (Dumond, 1937 ▸), and as a result the throughput flux is reduced. The reduction presently can be quantified from the profiles shown in Fig. 2 ▸. The comparison shows that flux densities for the four-reflection geometry was 5% of that for the two-reflection geometry.

## 5. Transport of the intensity after the grating

Talbot interferometer data were simulated by transporting the electric field, and thus the intensity, as a function of *z*, first transmitted through the grating and then propagated from the grating to the scintillator. The transmission and propagation was carried via equation (1) (Momose *et al.*, 2003 ▸; Guigay, 1971 ▸),

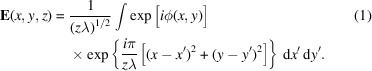

**Figure 3:**
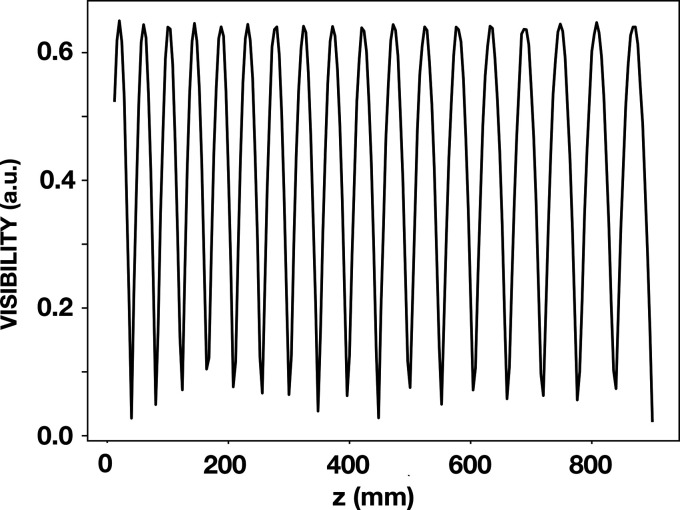
Simulated fringe visibility without the incorporation of a CCF. The results were obtained by propagating a plane wave incident on the checkerboard grating at 8.500 keV. The ordinate is the amplitude of the second-order term in the Fourier transform of the intensity normalized to the zeroth order. Absorption in the gold squares of the grating was incorporated. An effective *z* scale to account for stretching due to a spherical incident wave was also incorporated. The line connects calculated results at points at 4 mm separation, in order to correspond to *z* locations at which beamline data were obtained.

Here, *y* is the vertical transverse coordinate, *x* is the horizontal transverse coordinate, ϕ is a phase shift of π at the checkerboard pads and zero between pads, and λ is the X-ray wavelength. This equation was applied, and results for the two-dimensional partial Talbot effect were found to be in agreement with prior results (Weitkamp *et al.*, 2006 ▸; Zanette, 2006 ▸). That is, there was agreement for the Talbot carpet. The resulting intensities as a function of *z* were then Fourier transformed in two dimensions and the second-order Fourier coefficient was computed along both the vertical and horizontal directions. This produced the result shown in Fig. 3 ▸ which applies to a spherical incident wave. Here the *z*-scale was stretched to account for a spherical incident wave instead of a plane wave (Weitkamp *et al.*, 2006 ▸). The results in Fig. 3 ▸ do not account for the partial coherence of the beam incident on the grating. Ray tracing was carried out in order to simulate such partial coherence.

## 6. *SHADOW* ray tracing and phase space results

As part of the present study, extensive ray tracing was carried out via the *ShadowOui* program available from the *OrAnge SYnchrotron Suite* (*OASYS*) (Rebuffi & Sanchez del Rio, 2016 ▸, 2017 ▸). The second crystal in the DCM was detuned to reduce the throughput by 50% in order to suppress Si 333 harmonic radiation. This setting mimicked the procedure used during the measurements. Ray tracing was carried out downstream of the asymmetric crystal reaching to the grating.

**Figure 4:**
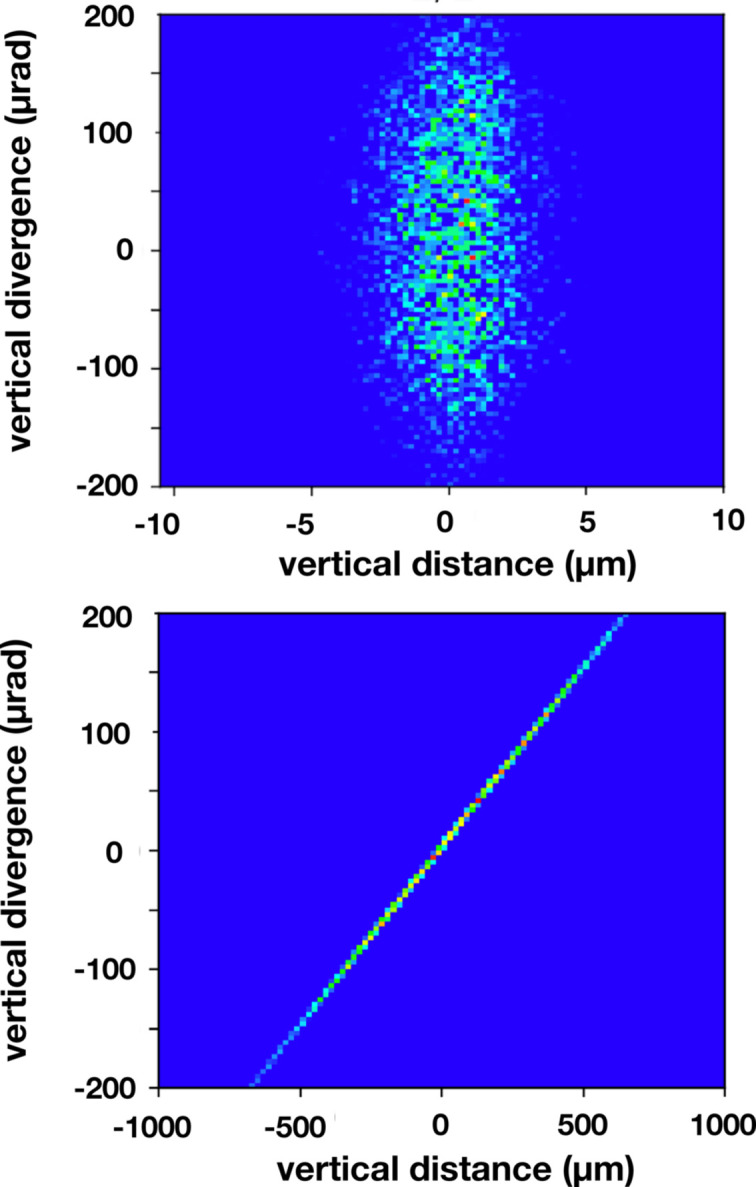
Phase space results obtained by ray tracing for the four-reflection geometry at 8.500 keV. The abscissa of each plot is the vertical coordinate of each ray, and the ordinate is the vertical divergence of each ray. The upper panel is at the effective source location which is 3.32 m upstream of the grating, and the lower panel is at the grating. These results illustrate how the effective source location was ascertained. That is, the upstream distance was varied until the symmetric diagram shown in the upper panel was the result.

The ray-tracing results were used to simulate an effective source as shown in Fig. 4 ▸ which shows (vertical distance)/(vertical divergence) phase spaces both at the grating in the lower panel as well as at the deduced effective source located upstream of the grating and shown in the upper panel. These results show the divergence of rays at specific vertical positions. A symmetric phase space diagram, such as would be used to represent the bending magnet source, for example, when propagated downstream spreads to larger vertical distances but retains the same spread in divergence. So, for example, rays with the largest divergence spread to the largest vertical position. With the ray-tracing result at the grating, it is a simple matter to reverse this propagation and determine at which distance upstream the phase space diagram becomes symmetric. This is how we obtained the effective source position. The effective source determined in this way is shown in the upper panel in Fig. 4 ▸ and was found to occur at 3.32 m upstream, very near the asymmetric crystal.

The van Cittert–Zernike theorem was then applied to the effective source.

## 7. van Cittert–Zernike theorem

The CCF, denoted in equation (2) as μ, between two points in the observation plane separated by Δ*y* vertically and by Δ*x* horizontally was calculated as per the van Cittert–Zernike theorem (Goodman, 1985 ▸) for an incoherent source. The applicable equation, to within a phase factor, is 

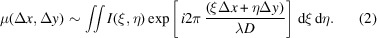

Here, *I*(ξ, η) is the intensity in the source plane at transverse coordinates ξ and η, and *D* is the distance between the source and the grating. Since the grating was a checkerboard, the CCF that equals the absolute value of μ could be obtained as a function of both vertical and horizontal directions. We first apply this equation at the grating and note that it is a Fourier transform with frequencies given by *f* = Δ*y*/λ*D* for the vertical direction and Δ*x*/λ*D* for the horizontal direction.

Interference occurs downstream of the grating, which can be viewed as a beam-splitter and which relates the Δ*y* and *z* scales as (Pfeiffer *et al.*, 2005 ▸) 

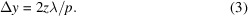

This procedure can also be described as projecting the source profile (Weitkamp *et al.*, 2006 ▸) or as the application of a transfer function (Shi *et al.*, 2022 ▸). The net result is that the CCF can be related to *z* settings for the measurements via the Fourier transform frequency, that is, by applying *z* = *pDf*/2.

**Figure 5:**
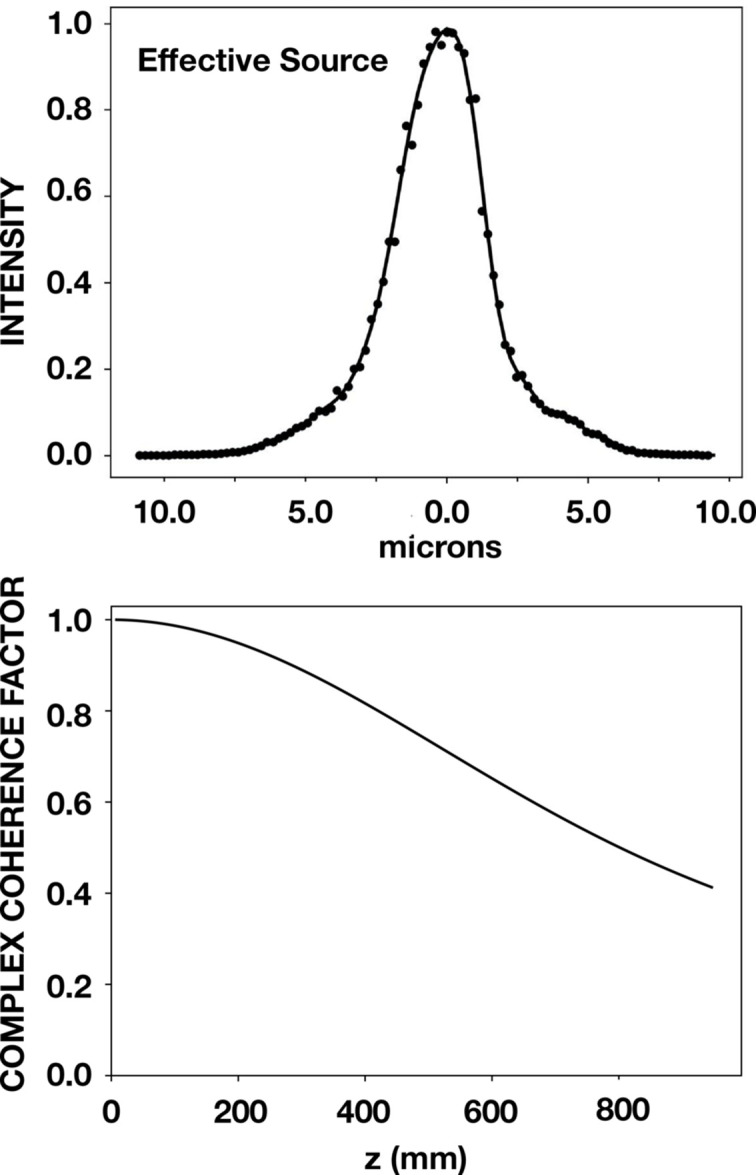
Effective source and CCF. The phase space results at *D* = 3.32 m upstream of the grating were projected to yield the effective source profile shown in the upper panel. The effective source size, *S*, here taken to be the FWHM of the profile, is *S* = 3.2 µm. The effective source projects a divergence of *S*/*D* = 0.96 µrad at a point in the grating. The lower panel shows the CCF function obtained from the effective source as described in the text.

The effective source shown in the upper panel of Fig. 4 ▸ was projected onto the vertical *y*-axis to yield the profile shown in the upper panel of Fig. 5 ▸. The ray-tracing results, shown as points, were fit to a polynomial function. This function was then Fourier transformed, and via the frequency the CCF function shown in the lower panel of Fig. 5 ▸ was obtained.

To determine a value for the SCL, the *z* value for a decrease of exp(−1/2) = 0.606, denoted as z*, was applied in equation (3). This procedure mimics that for a Gaussian as given in 

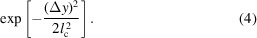

Here, a value of exp(−1/2) is obtained for *l*_c_ = Δy*, and Δy* is obtained from equation (3) with application of z*. The net result is an SCL value given by 2λz*/*p*. A value of 38.9 µm was obtained for the SCL in the case of Fig. 5 ▸.

## 8. Comparison with beamline data

**Figure 6:**
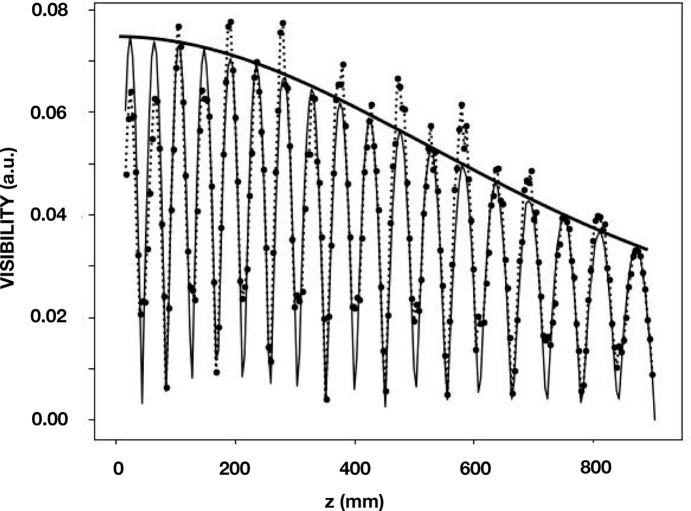
Visibility results at 8.500 keV for the four-reflection geometry in the vertical direction. Shown are (i) beamline data obtained by Talbot interferometry as points together with dashed lines that connect these points, (ii) simulated Talbot interferometry as a solid light line, and (iii) the CCF shown in the lower panel of Fig. 5 ▸ as a solid heavy line.

The effect of partial coherence was simulated by means of the CCF shown in Fig. 5 ▸. The product of this function and the calculated visibility shown in Fig. 3 ▸ is shown in Fig. 6 ▸. This figure also shows the data points that were obtained by applying *wavepy2* to the beamline data. The overlay of the simulation and the data points demonstrate a good agreement.

## 9. Four-reflection – 8.500 keV horizontal

**Figure 7:**
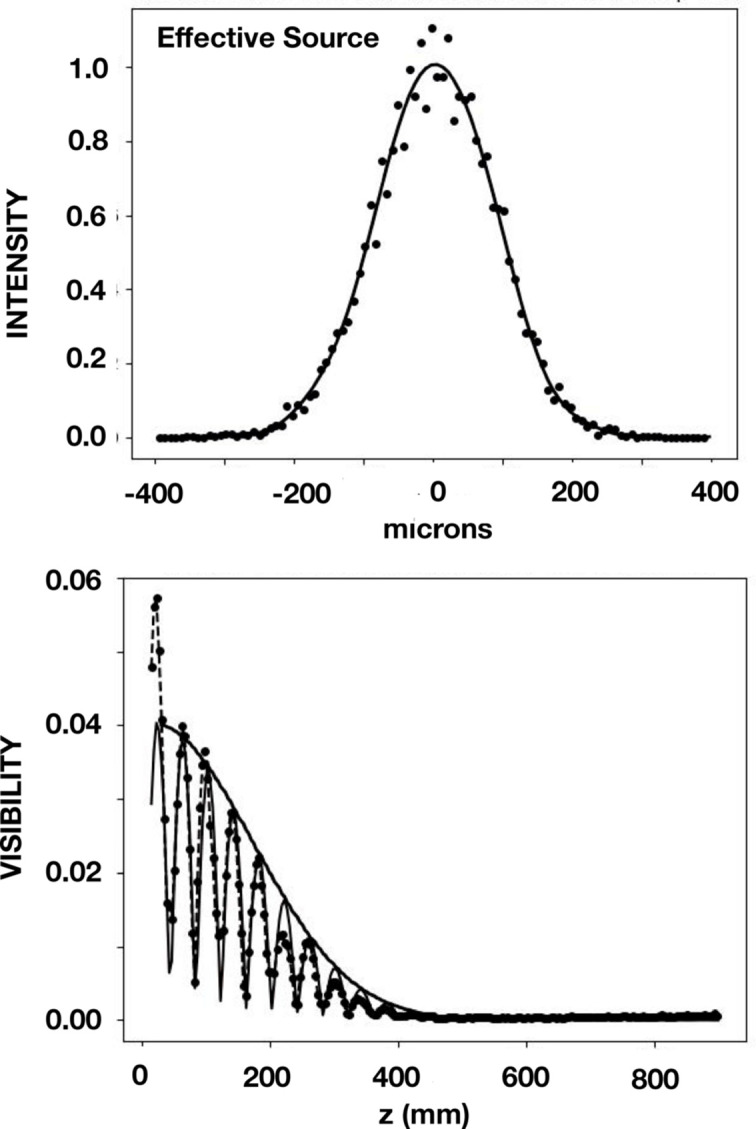
Effective source and interferometry results at 8.500 keV for the four-reflection geometry in the horizontal direction. The effective source is shown in the upper panel. In the lower panel are shown (i) beamline data obtained by Talbot interferometry as points together with dashed lines that connect these points, (ii) simulated Talbot interferometry as a solid light line, and (iii) the CCF calculated from the profile shown in the upper panel as a solid heavy line.

The same procedures were followed for the horizontal direction. The results for the four-reflection geometry at 8.500 keV in the horizontal direction are shown in Fig. 7 ▸. The distance *D* between the effective source and the grating was found by ray tracing to be 35.5 m. That is, the effective source was the bending magnet itself. This is expected for a heat bump limited to the vertical plane. A value of 9.7 µm was found for the SCL in this case.

## 10. Direct – 8.500 keV

**Figure 8:**
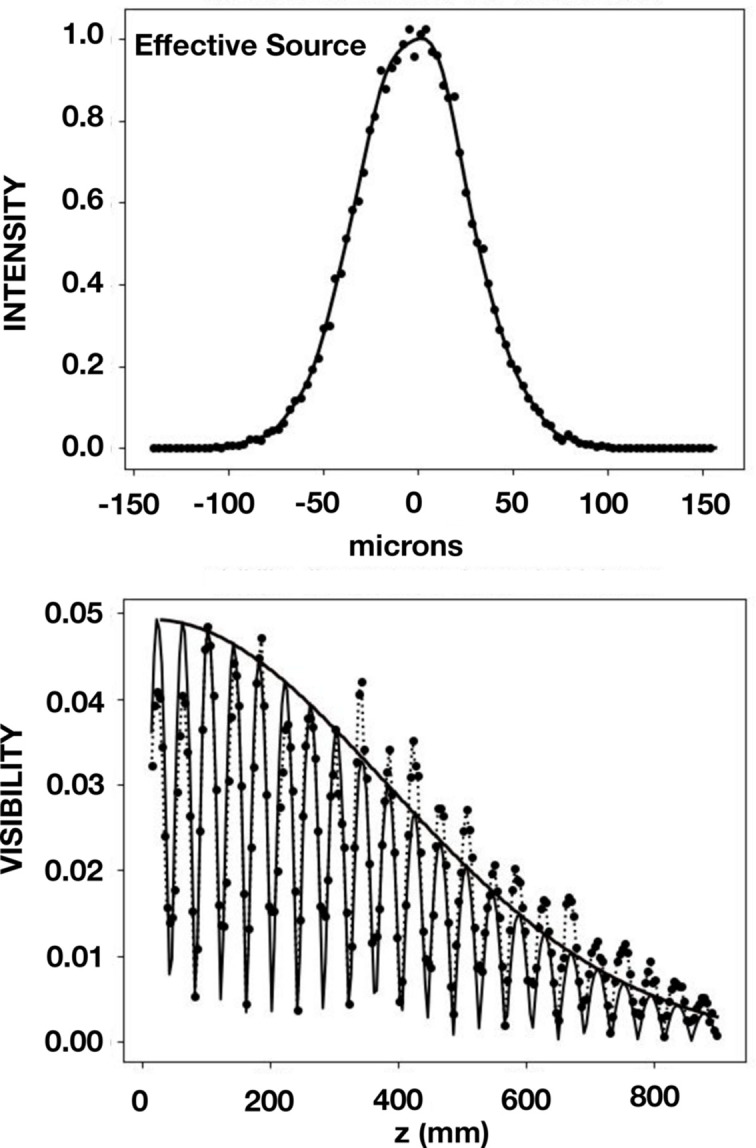
Results in the vertical plane at 8.500 keV for the two-reflection geometry which is for a beam directly exiting the DCM. In the upper panel is shown the result obtained with the *SHADOW* ray-tracing program. It shows the profile of the intensity versus vertical distance at the effective source located 28.8 m upstream of the grating. In the lower panel are shown (i) beamline data obtained by Talbot interferometry shown as points together with dashed lines that connect these points, (ii) simulated Talbot interferometry as a solid light line, and (iii) the CCF, calculated from the profile shown in the upper panel, as a solid heavy line.

**Figure 9:**
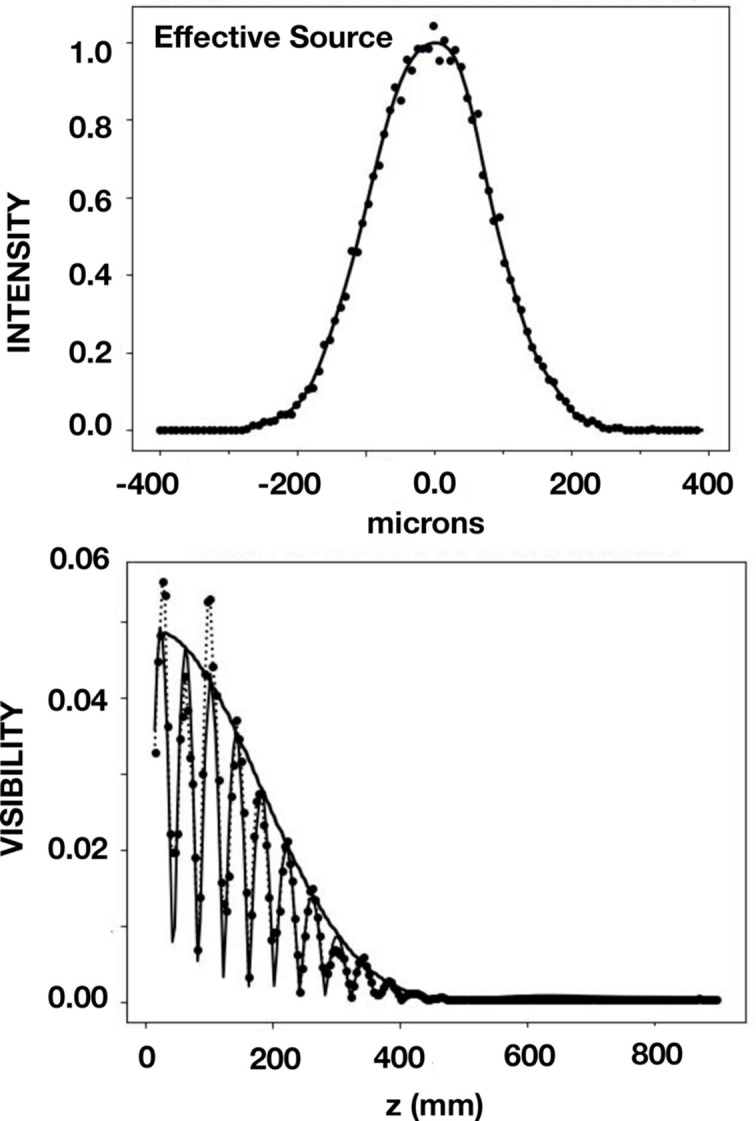
Results in the horizontal plane at 8.500 keV for the two-reflection geometry which is for a beam directly exiting the DCM. In the upper panel is shown the result obtained with the *SHADOW* ray-tracing program. It shows the profile of the intensity versus horizontal distance at the effective source, that is, at the bending magnet located 35.5 m upstream of the grating. In the lower panel are shown (i) beamline data obtained by Talbot interferometry as points together with dashed lines that connect these points, (ii) simulated Talbot interferometry as a solid light line, and (iii) the CCF, calculated from the profile shown in the upper panel, as a solid heavy line.

Measurements and simulations were also made for a beam directly exiting the DCM. In the vertical plane a value of *D* equal to 28.8 m was found, which is shorter than the distance to the bending magnet. By introducing a heat bump of 20 nm on the first crystal in the DCM, a value of 29 m for *D* was also obtained by means of ray tracing. The results are shown in Fig. 8 ▸. A value of 22.6 µm was found for the SCL in the vertical direction. We note that this reveals a gain of 76% for the SCL of the four-reflection case over the two-reflection case, albeit with a factor of 20 reduction in flux density. Results in the horizontal plane are shown in Fig. 9 ▸. A value of *D* equal to 35.5 m was found for both the data and the simulations. This distance corresponds to the distance from the bending magnet, confirming again that the heat bump was only effective in the vertical plane.

## 11. Results at 8.200 keV

A full set of measurements were also made at 8.200 keV for which the exit angle is 0.88°, that is, roughly twice that at 8.500 keV. The Talbot interferometry measurements could also be well simulated with the 50% detuning setting implemented at the start of *z*-scans during the data taking. The FWHM of the effective source was 8.0 µm, a considerably larger value than was obtained at 8.500 keV, namely 3.2 µm. This larger FWHM resulted in an SCL value of 19.7 µm, a value considerably reduced from the 38.9 µm value for 8.500 keV. The results at 8.200 keV are summarized in Table 1 ▸ along with the results at 8.500 keV that were presented above. Notable here is the reasonable agreement between all values of *D* and between all values of the SCL in the horizontal direction. Of particular note is the reduced value of 23.4 µm for the SCL in the vertical direction for the four-reflection configuration at 8.200 keV. We conclude that the four-reflection geometry must be at quite low grazing exit conditions to be effective in increasing the SCL.

## 12. Summary

In summary, in comparison with a beam exiting a DCM, the spatial coherence length was found to have an increased value for a (+ − − +) four-reflection geometry in which the fourth reflection is asymmetric and configured at grazing exit. Detailed Talbot interferometry results obtained at beamline 1-BM at the Advanced Photon Source with a checkerboard grating are presented together with extensive ray-tracing simulations at 8.500 keV and 8.200 keV. Results in both the vertical and the horizontal directions are reported not only for the four-reflection case but also for the beam directly exiting the DCM. An effective source located very near the asymmetric crystal resulted from the ray tracing, leading to a determination of the CCF which caused the amplitude of Talbot fringes to decrease with increasing distance between the grating and detector. The CCF was determined without the assumption that it was a Gaussian function. A value of 38.9 µm was obtained for the spatial coherence length at 8.500 keV for a Si 111 reflection with an asymmetry angle of 13.1°. This corresponded to an exit angle of 0.41°. This spatial coherence length was 76% larger than that obtained for the beam that exited the DCM.

## Figures and Tables

**Figure 10:**
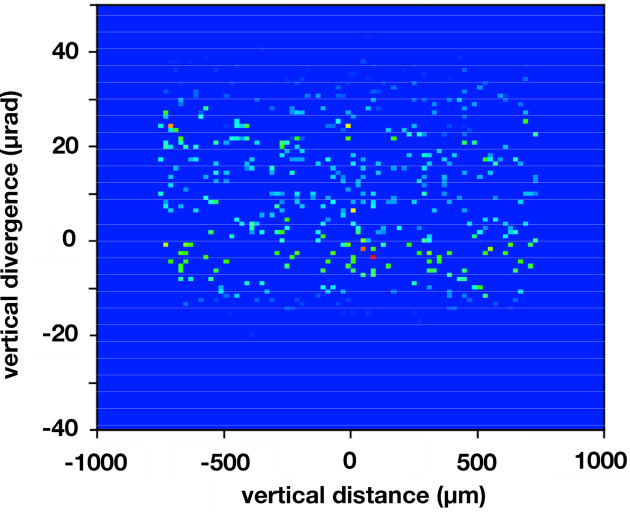
Ray-tracing results for the phase space at the grating position for the same experimental arrangement as shown in Fig. 1 ▸ except that the fourth crystal is set to diffract at grazing incidence instead of at grazing exit. The corresponding SCL is only 3.0 µm.

**Table 1:** Results summary

Energy (keV)	Four- or two-reflection	Vertical or horizontal	*D* (m)	SCL (µm)
8.5	4	Vertical	3.32	38.9
8.5	2	Vertical	28.8	22.6
8.5	4	Horizontal	35.5	9.7
8.5	2	Horizontal	35.5	9.8
8.2	4	Vertical	3.34	19.7
8.2	2	Vertical	29.0	23.4
8.2	4	Horizontal	35.5	9.8
8.2	2	Horizontal	35.6	10.1

**Table 2:** Comparison with other means to increase the spatial coherence length

Method	Effective source height, σ (µm)	Effective source distance, *D* (m)	λ*D*/πσ (µm)	Intensity (10^6^ rays)
Fourth crystal at grazing exit	1.80	3.32	43	263
Fresnel zone plate focused on pinhole	1.48	3.26	51	5
Propagation after DCM	31	49.7	37	932

## Appendix A Comments on grazing exit versus grazing incidence

At grazing incidence the beam footprint and exit beam cross section can be increased over that for a symmetric reflection. Concomitant is a decreased exit divergence, that is, the exit beam is more collimated. The grazing incidence geometry was also simulated presently by means of ray tracing. The (vertical distance)/(vertical divergence) phase space result shown in Fig. 10 ▸ was obtained at the grating position at 8.500 keV. We note that this phase space result is in stark contrast to that for the grazing exit geometry shown in the lower panel of Fig. 4 ▸. The divergence at the center of the grating evident in Fig. 10 ▸ is α = 25 µrad FWHM, and, when applied to (Brauer *et al.*, 1995*b* ▸)

yields a value of only 3.0 µm for the SCL. A significant aspect of the present work is that one must consider not only the usual 2-d Dumond phase space of wavelength and divergence but also include position as a third axis. With this included by means of *SHADOW*, the grazing exit geometry does produce a wide overall vertical exit divergence, as shown in the lower panel of Fig. 4 ▸, but the divergence at a single vertical position is much reduced to 3 µrad. As per the van Cittert–Zernike theorem, the divergence that sets the spatial coherence length is the divergence at a single point in the object field. For the grazing incidence geometry, although the overall divergence is roughly an order of magnitude smaller, a vertical divergence of 25 µrad pertains at all points in the object field which results in a significantly shorter coherence length compared with the grazing exit case.

## Appendix B Comparison with other ways to increase the spatial coherence length

In this Appendix we compare our method with two other means to increase the spatial coherence length. These two means are: (i) use a focusing optic to focus on a secondary source, and (ii) simply allow the beam to propagate in vacuum. *SHADOW* simulation results are compared and contrasted with the four-reflection case as summarized in Table 2 ▸. In both cases (i) and (ii), the only crystals in the beam path were in the DCM.

For case (i) we simulated a phase-type Fresnel zone plate (FZP) focused on a circular aperture. This aperture was located at the same downstream position as was the grazing exit crystal, 32.2 m, and the distance from the aperture to the object plane, *i.e.* the plane of the grating, was also set to be the same, 3.32 m. The diameter of the FZP was taken to be 1.00 mm, which is near a realistic maximum for a FZP as set by lithography fabrication methods. The outer-most zone width was 175 nm, and the focal length was 120 cm. For the purpose of making the comparisons summarized in Table 2 ▸, we treated all beams as having a Gaussian shape. This resulted in a slightly larger (by 4 µm) SCL of 43 µm for the present four-bounce grazing exit arrangement. In the case of the FZP, the waist at the focus was 34 µm FWHM. A value of 34 µm was set for the diameter of the secondary source aperture. The upstream distance of the effective source, *D*, was determined in *SHADOW* to be 3.26 m, and the effective source size value for σ was 1.48 µm. For comparison purposes the SCL applicable in the case of a Gaussian source, namely, λ*D*/2πσ (Marathe *et al.*, 2014 ▸), is listed in Table 2 ▸. This value was 51 µm, a value somewhat comparable with the grazing exit four-reflection case presented above. We note that the parameters for the FZP and secondary source were chosen to make SCL come out roughly comparable with the grazing exit case. The final column in Table 2 ▸ lists a simulated intensity. The intensity found in all three cases summarized in Table 2 ▸ was for one million rays emanating from the bending magnet in *SHADOW* simulations. The intensity for the FZP plus a secondary source case was found to be only 5 as compared with a value of 263 in the case of the grazing exit crystal presented above. This amounts to reduction of a factor of 53 in intensity, which is a relative disadvantage. There is the additional disadvantage of having to block scattering from the edges of the secondary source.

For case (ii) we simulated a beam that was allowed to propagate an additional distance from the DCM. For comparison purposes we set this additional distance to be such that the SCL, 37 µm, was again roughly comparable with the value obtained for the grazing exit crystal case presented above. In this case the σ value for the effective source size was 31 µm, and the effective source distance, *D*, was 49.7 m. As listed in Table 1 ▸ for the two-beam vertical case a value of *D* equal to 29 m was measured in the case of a beam coming directly from the bending magnet which resulted in an SCL of 23 µm. As summarized in Table 2 ▸, an additional propagated distance of 21 m is required to increase this value to 37 µm, albeit with an improved intensity by a factor of 3.5 over that of the grazing exit crystal case. The disadvantage of the pure propagation case is the need for a beamline that is longer by 21 m.
